# Sustained Successful Treatment of Two Phenotypically Different Cases of Genital Papular Acantholytic Dyskeratosis

**DOI:** 10.7759/cureus.90583

**Published:** 2025-08-20

**Authors:** Sarah M Johnston, Vrinda Bajaj, Aparna Bhat, Paul Barrett, Sachinta G Wijesiri

**Affiliations:** 1 Department of Dermatology, Queen Elizabeth University Hospital, Glasgow, GBR; 2 Department of Dermatology, University Hospital of North Durham, Durham, GBR; 3 Department of Dermatology, Barking, Havering and Redbridge University Hospitals NHS Trust, Romford, GBR; 4 Department of Pathology, University Hospital of North Durham, Durham, GBR; 5 Department of Obstetrics and Gynaecology, University Hospital of North Durham, Durham, GBR

**Keywords:** anogenital papules, genitocrural papules, gpad, papular acantholytic dyskeratosis, vulval papules

## Abstract

Genital papular acantholytic dyskeratosis (GPAD) is a rare, recurrent condition. It is characterised by skin coloured or whitish papules which may coalesce to form plaques affecting the genitocrural or anogenital skin in women and men. Histological features include hyperkeratosis, focal parakeratosis with acantholytic and dyskeratotic cells. It is recognised as a distinct clinicopathological entity localised to genital skin, but there are increasing suggestions of genetic links with Hailey-Hailey disease and Darier's disease. Heat, friction, and hormonal influence may play a role in aetiology. We present two patients with different phenotypical presentations who have had effective treatment sustaining remission. Corticosteroids are widely reported as an ineffective treatment, but our first patient had a rapid response to oral prednisolone without recurrence. The second case supports the evidence that surgical excision is an effective option for localised disease. Both patients presented via circuitous routes. Diagnosis is often delayed and likely underestimated. Our first patient developed the condition after surgical menopause, which may add to evidence of hormonal influence. We highlight that GPAD is an important differential to consider for vulval, anogenital, or genitocrural papules.

## Introduction

Papular acantholytic dyskeratosis (PAD) is a rare, chronic or recurrent benign dermatosis affecting genitocrural skin [1]. In terms of clinical features, it is characterised by persistent skin coloured or whitish papules which may coalesce to form plaques affecting the genitocrural or anogenital skin in women and less commonly in men [2]. GPAD can be asymptomatic or associated with pruritus or burning [3].

Histological features include hyperkeratosis, focal parakeratosis with acantholysis (separation of keratinocytes in the epidermis, due to loss of adhesion between cells), and dyskeratotic cells (premature abnormal keratinisation in the epidermis). It is recognised as a distinct clinicopathological entity localised to genital skin, without a family history of a similar condition. However, pathogenesis is not clear; there are increasing suggestions of genetic links with Hailey-Hailey disease (HHD) and Darier's disease (DD) [4-8]. Heat, friction, and hormonal influence may play a role in aetiology [9,10].

Diagnosis of GPAD is often delayed as it is a rare condition. Clinical differential diagnosis includes viral condyloma and plane warts, herpes, molluscum, milia, and fordyce spots. Histopathological differential includes HHD, DD, transient acantholytic dyskeratosis, and warty dyskeratoma. Therefore, accurate clinicopathological correlation is required for diagnosis [5,7]. It is important to consider GPAD as an early differential for genital papules. Misdiagnosis as an infection or sexually transmitted disease can lead to unnecessary patient distress. Management is challenging as treatment options are currently based on case reports and case series. We present two different phenotypic cases treated differently to add to the existing literature on management options.

## Case presentation

Case 1

A 59-year-old woman presented to Genitourinary Medicine (GUM) with a nine-month history of pruritic papulovesicular eruption in the genitocrural area. Eruption persisted despite empirical treatment for genital herpes with aciclovir, followed by a trial of potent topical steroids and topical antifungals. Herpes simplex and varicella zoster swabs were negative. GUM referred to Gynaecology for biopsy due to diagnostic uncertainty. Dermatology opinion was sought in light of her histology and progression of her eruption. Her past medical history included quiescent cutaneous lichen planus and hysterectomy with oophorectomy for dysmenorrhoea. There was no family history of similar symptoms or dermatological disease.

**Figure 1:**
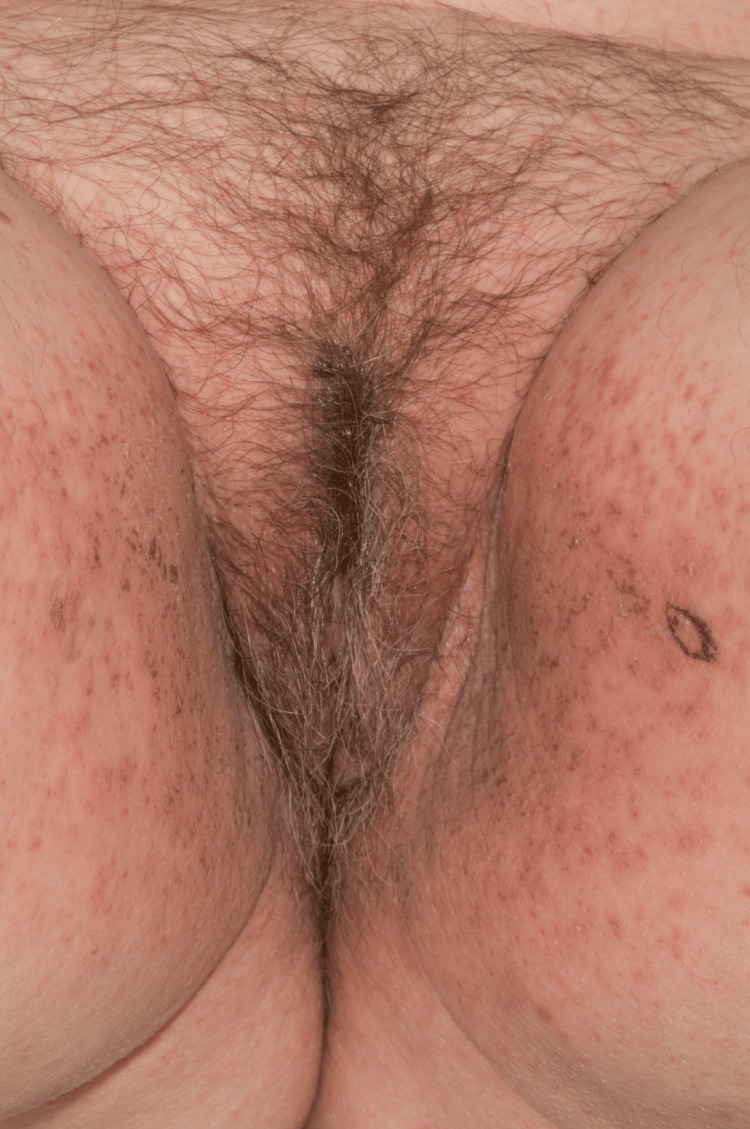
Red, purple papules and pseudovesicles on labia majora and upper inner thighs at presentation (Case 1)

**Figure 2:**
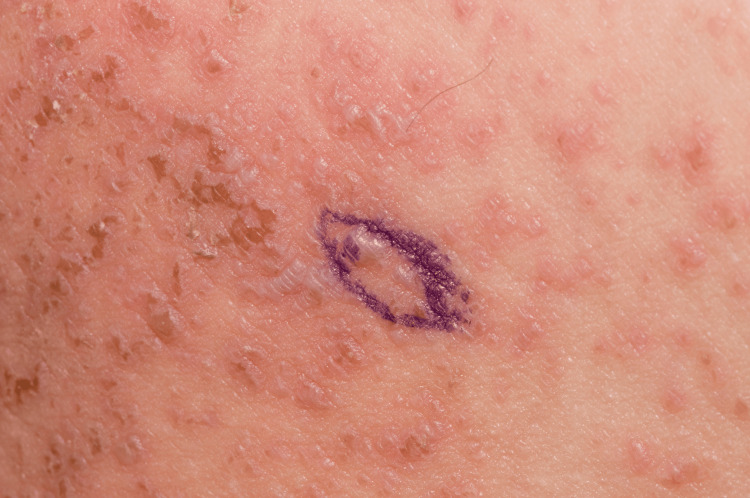
Close-up of papules on left upper inner thigh at presentation (Case 1)

On examination, she had a red-purple papular and pseudovesicular eruption of her labia majora with extension onto her upper inner thighs (Figures 1, 2).

She later developed a discrete eruption of violaceous flat-topped papules with Wickham’s striae. Histology from a wrist papule confirmed clinical suspicion of recurrence of lichen planus.

**Figure 3:**
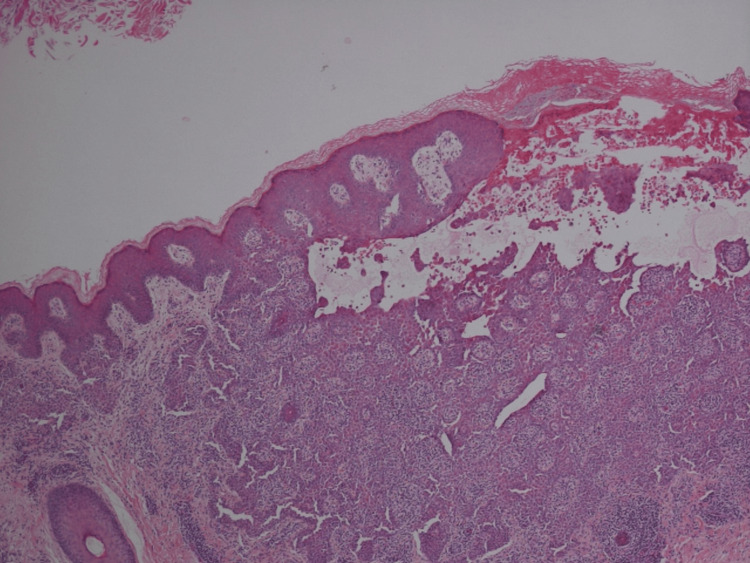
Incisional biopsy demonstrating acantholysis with hyperkeratosis and parakeratosis (haemotoxylin and eosin, magnification x40) (Case 1)

**Figure 4:**
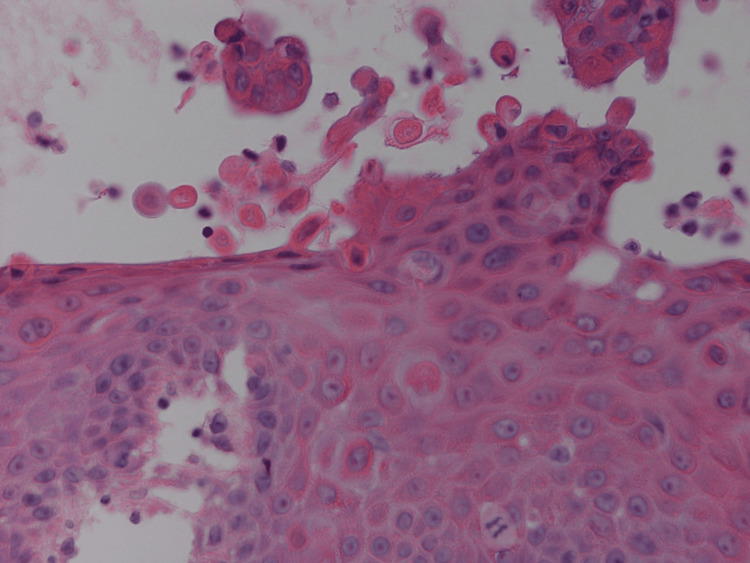
Higher power view of dyskeratosis with corp ronds in the upper epidermis and grains toward the stratum corneum (haemotoxylin and eosin, magnification x200) (Case 1)

Initial biopsy of labia majora showed epidermal acantholysis, numerous dyskeratotic cells with grains and corp ronds (Figures 3, 4). A second biopsy from the inner thigh demonstrated consistent histological findings, and immunofluorescence was negative.

**Figure 5:**
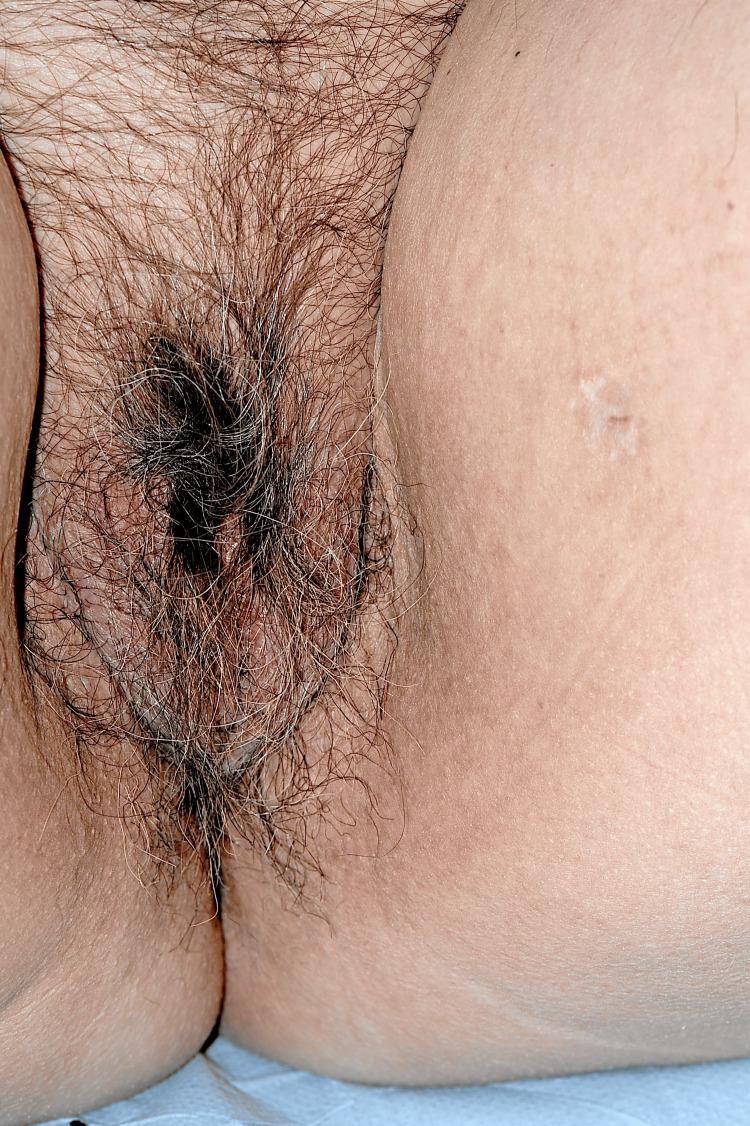
GPAD resolved following two month weaning course of oral corticosteroids (Case 1) GPAD: genital papular acantholytic dyskeratosis

Ultra-potent topical steroid eased her symptoms, but papules persisted. She was commenced on oral corticosteroids (40 mg weaning course) to manage her co-existing symptomatic lichen planus. Within two months, her papular genitocrural eruption resolved (Figure 5). She has had no recurrence in eight years.

Case 2

A 38-year-old woman was referred to Gynaecology via the urgent cancer suspected pathway with a nine-month history of localised, recurrent vulval ulceration with associated discomfort and pruritus. Treatment with potent topical steroids and topical anti-fungal in primary care was ineffective. She had no relevant past medical history or medications. There was no family history of any skin conditions.

**Figure 6:**
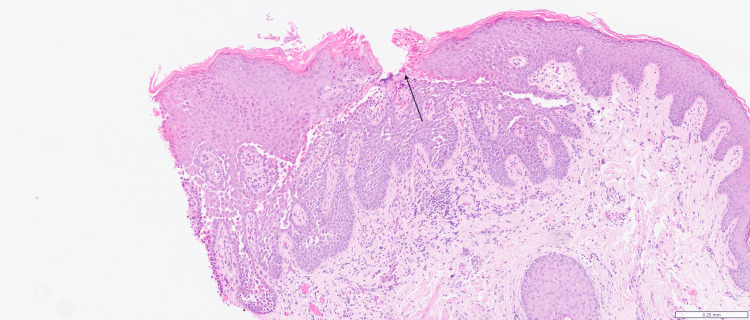
Vulval punch biopsy demonstrating acantholysis and dyskeratosis. Grains in epidermis highlighted by arrow (haemotoxylin and eosin, magnification x40) (Case 2)

**Figure 7:**
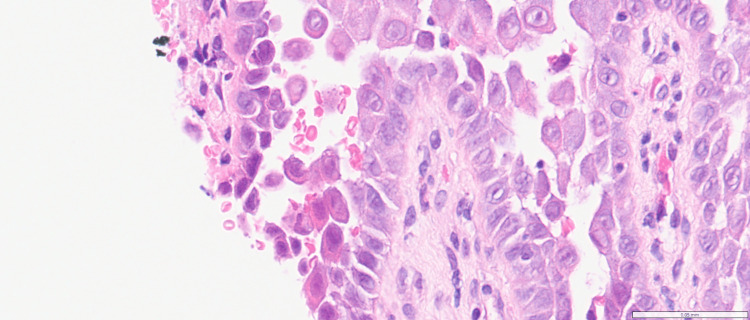
Higher power, focal area of acantholysis (haemotoxylin and eosin, magnification x200) (Case 2)

On examination, she had a 3 mm papule with central ulceration of the right labia majora. She was treated with an excisional punch biopsy, which was curative. Histology showed acantholysis and dyskeratosis (Figures 6, 7). Vulval multidisciplinary review with Dermatology, Gynaecology, and Pathology found the diagnosis to be consistent with focal GPAD. Telephone follow-up confirmed she has had no recurrence since excision three years ago.

Table 1 gives a summary of the two cases. 

**Table 1:** Summary of the cases

Parameters	Case 1	Case 2
Age	59 years	38 years
Duration of symptoms	9 months	9 months
Anatomical site	Bilateral labia majora and inner thighs	Right labia majora
Clinical appearance	Red-purple papules and pseudovesicular eruption	Skin coloured papule, with central ulcer
Symptoms	Pruritus	Pruritus, discomfort
Ineffective treatment	Mild/ moderate/potent topical steroid, topical antifungal (oral aciclovir when misdiagnosed)	Potent topical steroid, topical antifungal
Successful treatment	Ultra-potent topical steroid – improved symptoms, oral corticosteroids - resolution of eruption	Excision
Follow up	No recurrence in 8 years	No recurrence in 3 years

## Discussion

PAD was initially reported by Chorzelski et al. in 1985 as a distinct clinicopathological entity localised to genital skin [1]. It characteristically presents as an eruption of multiple skin coloured or whitish papules which may coalesce to form plaques affecting the genitocrural or anogenital skin in women and less commonly in men [2]. A third of patients are asymptomatic, while the majority experience associated pruritus or burning [3].

While its characteristic histological pattern resembles both HHD and DD, it has been recognised as a distinct entity. Although aetiology remains unclear, there is increasing evidence of genetic links. Case reports describe *ATP2C1* mutation associated with HHD and *ATP2A2* mutation with DD, and family history of both conditions in GPAD, suggesting this may be an allelic clinical entity or a clinical manifestation along a common disease spectrum [4-8]. The predilection for genital involvement postulates that heat, friction, and occlusion may be predisposing factors. Hormonal influence may also contribute to pathogenesis in cases of premenstrual exacerbation and a peripartum case with spontaneous resolution [9,10]. There are no other documented cases of GPAD associated with surgical menopause, which may add to these existing theories. Case reports of positive direct immunofluorescence suggest a role of immune dysfunction [10,11].

As it is a rare condition, management of GPAD is challenging: there is no standardised treatment protocol, and there is a lack of large-scale studies [12]. For widespread involvement, current topical treatments are focused on symptomatic management, including emollients, topical steroids, topical immunomodulators (calcineurin inhibitors), antifungals, retinoids, and vitamin D, which are reported to have variable responses. One case report describes no recurrence following treatment with topical diclofenac, another describes resolution with oral retinoid, while a third describes partial improvement [2,13,14]. Topical and oral corticosteroids are often first-line treatment [15,16]. In keeping with the literature, our first patient had symptomatic relief from topical steroids while papules persisted. Oral corticosteroids are widely reported as an ineffective treatment, with Chorzelski’s case widely referenced [1]. Lee et al. also report no improvement with a maximum dose of 15 mg prednisolone [17]. Our patient had a brisk response to a 40mg prednisolone weaning course and has had no recurrence.

Surgical treatment is currently the most effective treatment for limited involvement, with cases without recurrence following electrocoagulation or excision [3]. Cryotherapy and CO2 laser have also been described as effective treatments to ease symptoms and to induce remission [2,3]. In keeping with the literature, our second patient has had no recurrence in three years following excision.

## Conclusions

To the best of our knowledge, this is the only case describing complete resolution of GPAD with oral corticosteroids. Although adverse effects and flare on discontinuation may limit their use, this is an example of oral corticosteroids inducing sustained remission. Our second patient adds to existing evidence that surgical excision in isolated lesions is an effective treatment.

Both cases presented via circuitous routes with previous misdiagnoses. This highlights that GPAD can be a challenging and often delayed diagnosis. Our cases emphasise considering GPAD as a differential diagnosis for vulval or anogenital papules and add to the limited existing evidence of optimal treatment options.
